# Diversity and distribution of CO_2_-fixing microbial community along elevation gradients in meadow soils on the Tibetan Plateau

**DOI:** 10.1038/s41598-022-13183-4

**Published:** 2022-06-10

**Authors:** Haiyan Feng, Zhe Wang, Pengli Jia, Jingping Gai, Baodong Chen, Shikuan Wang

**Affiliations:** 1 School of Earth Sciences and Resources, China University of Geosciences, 29 Xueyuan Road, Beijing, 100083 China; 2 Jilin Institute of Geological Survey, Jilin, 961 Mingxi Road, Changchun, 130102 China; 3 College of Resources and Environmental Sciences, China Agricultural University, Key Laboratory of Plant-Soil Interactions, Ministry of Education, 2 Yuanmingyuan West Road, Beijing, 100193 China; 4 State Key Laboratory of Urban and Regional Ecology, Research Center for Eco-Environmental Sciences, Chinese Academy of Sciences, Beijing, 100085 China; 5 University of Chinese Academy of Sciences, Beijing, 100049 China

**Keywords:** Ecology, Environmental sciences

## Abstract

Soil CO_2_-fixing microbes play a significant role in CO_2_-fixation in the terrestrial ecosystems, particularly in the Tibetan Plateau. To understand carbon sequestration by soil CO_2_-fixing microbes and the carbon cycling in alpine meadow soils, microbial diversity and their driving environmental factors were explored along an elevation gradient from 3900 to 5100 m, on both east and west slopes of Mila Mountain region on the Tibetan Plateau. The CO_2_-fixing microbial communities were characterized by high-throughput sequencing targeting the *cbbL* gene, encoding the large subunit for the CO_2_-fixing protein ribulose 1, 5-bisphosphate carboxylase/oxygenase. The overall OTU (Operational Taxonomic Unit) abundance is concentrated at an altitude between 4300  and  4900 m. The diversity of CO_2_-fixing microbes is the highest in the middle altitude area, and on the east slope is higher than those on the west slope. In terms of microbial community composition, Proteobacteria is dominant, and the most abundant genera are *Cupriavidus*, *Rhodobacter*, *Sulfurifustis* and *Thiobacillus*. Altitude has the greatest influence on the structural characteristics of CO_2_-fixing microbes, and other environmental factors are significantly correlated with altitude. Therefore, altitude influences the structural characteristics of CO_2_-fixing microbes by driving environmental factors. Our results are helpful to understand the variation in soil microbial community and its role in soil carbon cycling along elevation gradients.

## Introduction

Microorganisms have large biomass, numerous species, diverse metabolism, and complex interactions^1^. As an important biological component of the soil, soil microbes are the main driving force of soil organic matter and nutrient cycles and are regulating the biogeochemical cycling^2–4^ and maintaining ecosystem functions^5,6^. Autotrophic microorganisms are widely distributed in different ecosystems, and assimilation of CO_2_ is a key microbial process in the carbon cycle of global ecosystems and plays an extremely important role in regulating the concentration of carbon dioxide in the atmosphere^7^. The autotrophic microorganisms in global terrestrial soils can capture 0.5–4.1% of the atmospheric CO_2_ and total 0.6–4.9 Gt C each year^8^.

The Calvin-Benson-Bassham (CBB) cycle is a CO_2_ fixation pathway commonly found in plants and algae in nature^9^. It is also a pathway for photoautotrophic microorganisms and almost all aerobic autotrophic microorganisms to fix CO_2_. The global terrestrial soil ecosystem can fix 0.68–4.9 pg of carbon annually through the CBB cycle, accounting for about 4% of the total annual CO_2_ fixation in terrestrial ecosystems^10^. At present, the research on microorganisms with carbon-fixing function genes is mostly concentrated in aquatic ecosystems^11,12^, and most of the researches on carbon-fixing microorganisms in terrestrial ecosystems are also concentrated in areas with specific ecological conditions. The most common ones are soils in large farmland areas^13,14^. In extreme environments such as low temperature, drought, and high UV, soil carbon-fixing microbial communities may play an important role in CO_2_ fixation but have rarely been investigated.

The composition, distribution, and diversity of soil microorganisms are the keys to studying soil ecological functions. At the same time, soil microbial diversity and composition are also affected by soil physical and chemical properties in different environments^15^. A variety of environmental factors change with the change of elevation gradient^4^, so altitude is an important factor to detect the interaction between the autotrophic microbial community and environmental factors^16^. Some studies have shown that with the increase of altitude, most mountain soil microbial diversity shows a monotonous decreasing trend^17–19^, while the soil microbial diversity in some mountainous areas has a nonlinear relationship with the altitude gradient, which is manifested as a unimodal/inverted unimodal or bimodal/inverted bimodal distribution pattern^20–22^. The Tibet Plateau is the highest altitude area globally, with an average altitude of more than 4000 m. It is called the third pole of the world^23^. Its grassland area is about 1.2 × 10^6^ km^2^, accounting for about 48% of the plateau's land area^16^. It is sensitive to climate change and the disturbance of human activities. The Tibetan Plateau is a hotspot for biodiversity research, but there are few studies on soil functional microbial communities, especially CO_2_-fixing microbial communities. In this study, high-throughput sequencing method was used to study the change characteristics of CO_2_-fixed microbial community along altitude gradient in alpine meadow, which will help to understand the role of soil carbon-fixing microbial community in the process of soil carbon cycling, and provide scientific basis for more accurate evaluation of soil carbon sequestration in alpine meadow.

## Results

### Soil physicochemical factors along the elevation gradient

Most tested soil parameters significantly changed with increasing elevations (Table S1). The organic carbon content, total nitrogen, total carbon, available phosphorus, moisture content of the soil samples showed a trend of first increasing and then decreasing with the increase of altitude. The overall trend was more pronounced on the eastern slope compared with the western slope. Soil pH showed no significant change with altitude. The sand content and clay content of the western slope are higher than that of the eastern slope, and their contents do not vary greatly with altitude.

### Diversity of CO_2_-fixing microbes

**Figure 1:**
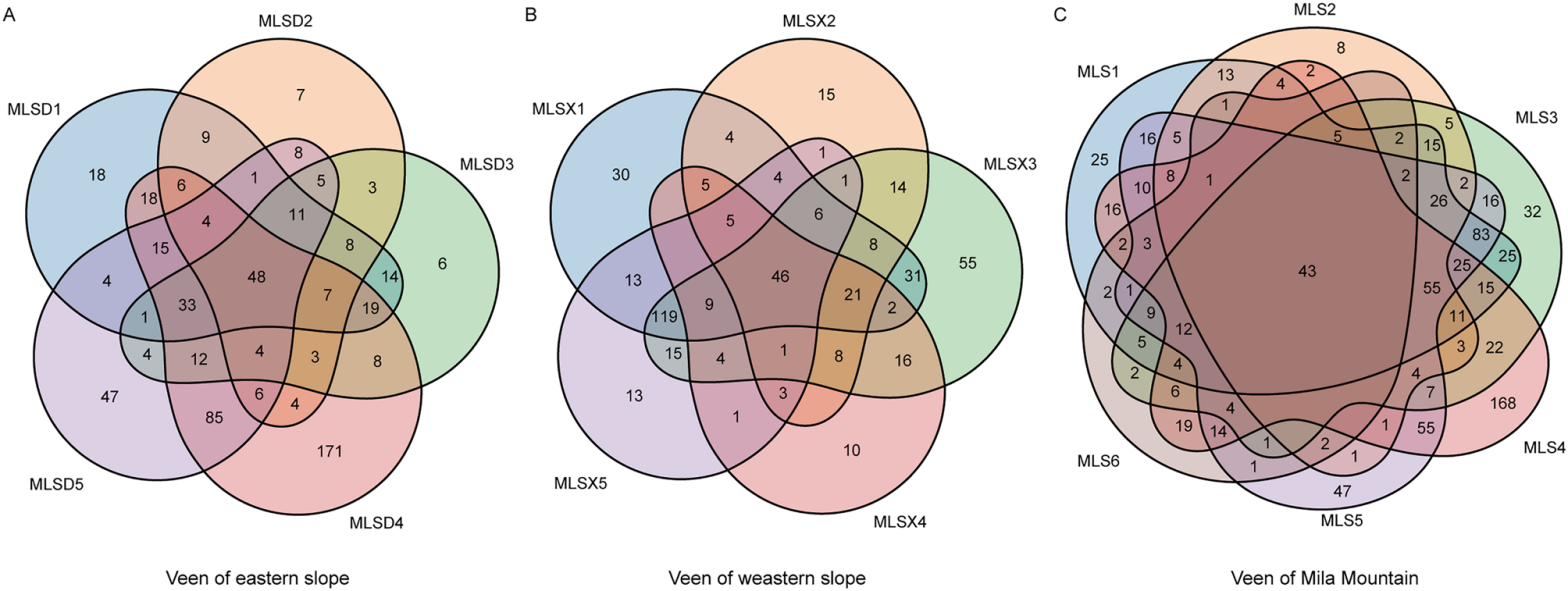
Venn diagram of OTU distribution along elevation gradients in Mila Mountain (**A** eastern slope; **B** western slope; **C** whole Mila Mountain).

According to the same elevation gradient, the samples of the same elevation on the east and west slopes were grouped. They numbered MLS1-5, corresponding to 3900–4100 m, 4100–4300 m, 4300–4500 m, 4500–4700 m, 4700–4900 m, respectively. MLS6 was separately corresponding to the peak. MLSD is the eastern slope of Mila Mountain, MLSX is the western slope of Mila Mountain. The numbers in the Venn diagram represent the number of OTUs. The number of OTU in the MLS4 is significantly higher than that in the other groups, with the corresponding altitude of 4500–4700 m. The eastern slope is mainly concentrated in MLSD4 and MLSD5 groups, corresponding to the elevation of 4500–4900 m, while the western slope has a large number of OTU in 4300–4500 m (Fig. 1).

Comparing the chao1 index of the east and west slopes of Mila Mountain, it is obvious that the eastern slope chao1 index of the MLS4 group is much higher than that of other altitude groups. Chao1 index of the eastern slope is generally higher than that of the western slope. Moreover, the east slope exhibited the highest species abundance on the 4500–4700 m zone, while the lowest is in the 3900–4100 m zone on the western slope (Table 1). It can be seen that the diversity of CO_2_-fixing microbes is the highest in the middle altitude area.

**Table 1:** Chao1 index and Shannon index of CO_2_-fixing microbes on Mila Mountain (mean ± SD).

	Sampling sites	Altitude (m)	Chao1	Shannon index
Eastern slope	MLSD1	3875	99.76 ± 35.54	3.37 ± 0.52
	MLSD2	4121	97.00 ± 6.06	2.40 ± 0.98
	MLSD3	4313	96.31 ± 26.78	2.60 ± 0.76
	MLSD4	4515	273.13 ± 39.50	4.87 ± 1.00
	MLSD5	4725	106.13 ± 66.44	1.88 ± 0.39
Mountaintop	MLS6	5020	90.08 ± 22.46	2.05 ± 0.78
Western slope	MLSX5	4846	81.96 ± 76.60	1.6110 ± 0.85
	MLSX4	4513	63.88 ± 26.3686	1.5500 ± 1.20
	MLSX3	4377	129.55 ± 80.63	3.1878 ± 1.31
	MLSX2	4145	87.86 ± 23.67	2.4345 ± 0.99
	MLSX1	3867	183.30 ± 72.18	2.6901 ± 0.83

Variation in Shannon index was similar to that of Chao1 index. In addition, the average Shannon index of the eastern slope is about twice that of the western slope (Table 1).

### Community structure of CO_2_-fixing microbes

**Figure 2:**
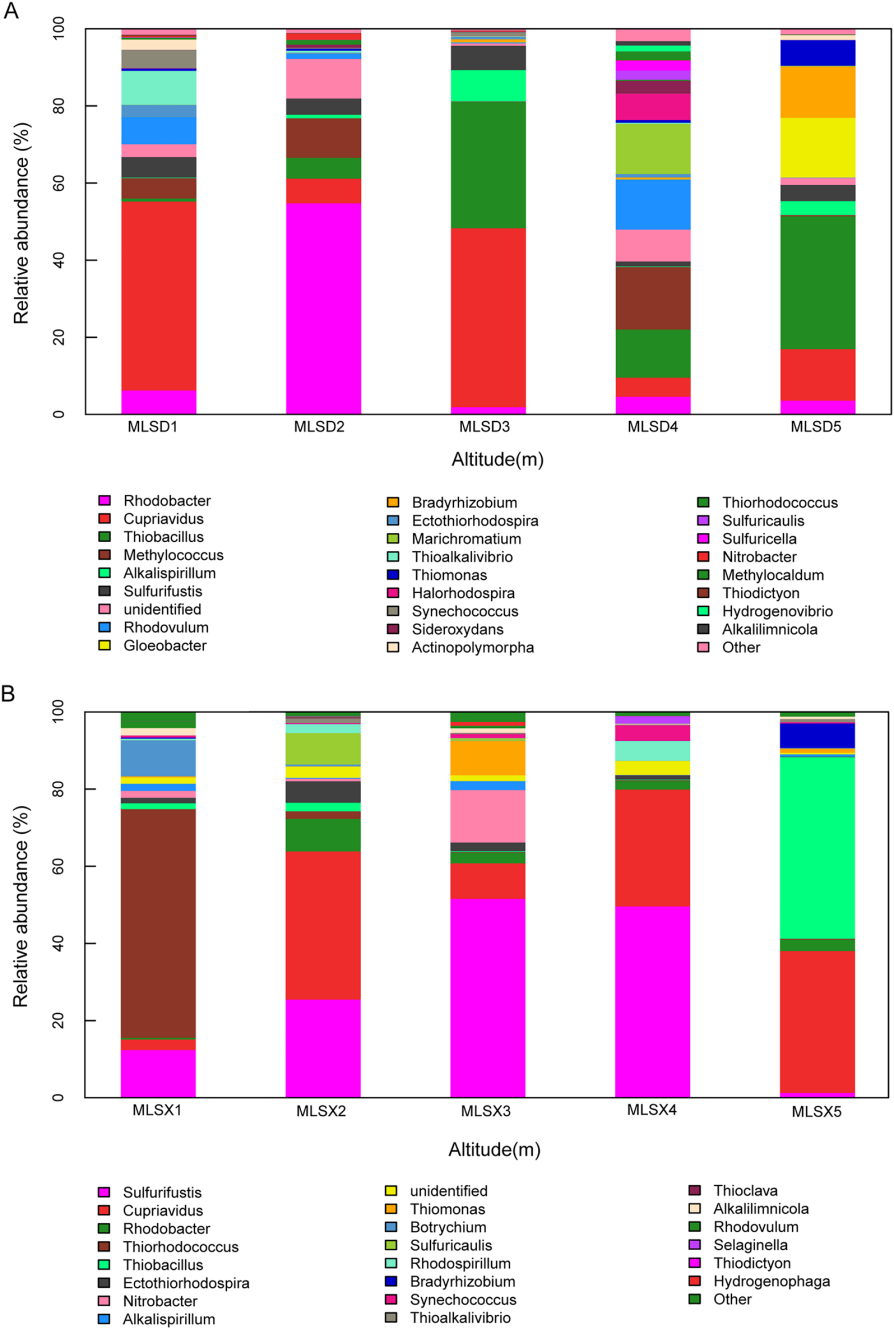

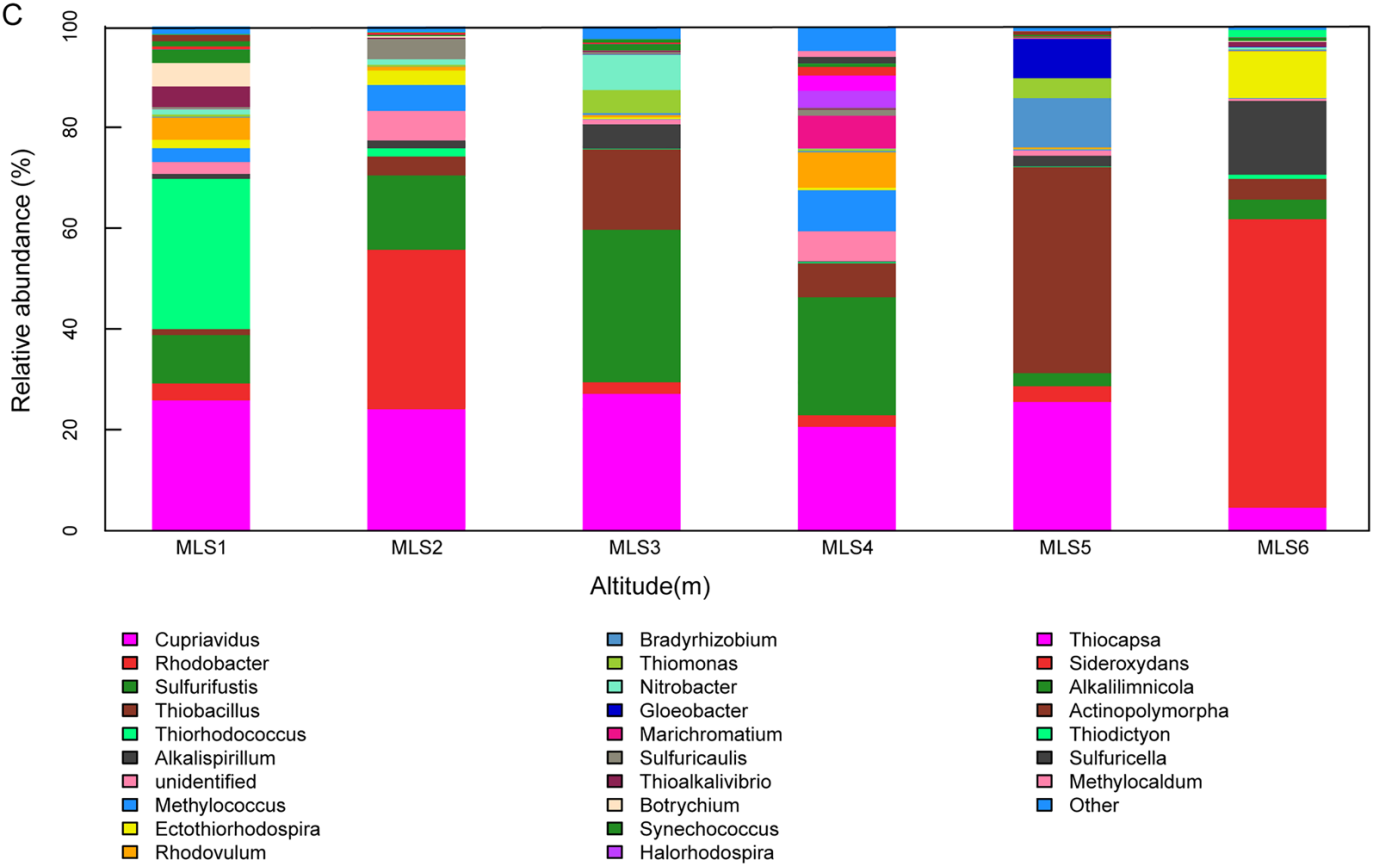
Community structure of CO_2_-fixing microorganisms along different altitude gradient zones (**A** eastern slope; **B** western slope; **C** whole Mila Mountain).

The composition of different microbial groups can be visually observed on the horizontal histogram (Fig. 2). The relative abundance of *Cupriaviidus* was the largest in the MLSD1 group, and the relative abundance of *Rhodobacter* was the absolute dominant in the MLSD2 and the MLS6 groups. The MLSD3 and MLSD5 group is mainly composed of *Cupriavidus* and *Thiobacillus*, while CO_2_-fixing microorganisms are evenly distributed in the MLSD4 group, and it is difficult to identify the dominant bacteria. *Rhodobacter* in the MLS6 group has the highest proportion, the MLSX5 group is composed of *Cupriavidus* and *Thiobacillus*. *Sulfurifustis* gradually decreases from MLSX4 to MLSX2 groups along the elevation. MLSX4, MLSX3, MLSX2 group also contain different proportions of *Cupriavidus*. MLSX1 group contains a large proportion of the unique *Thiorhodococcus*.

**Figure 3:**
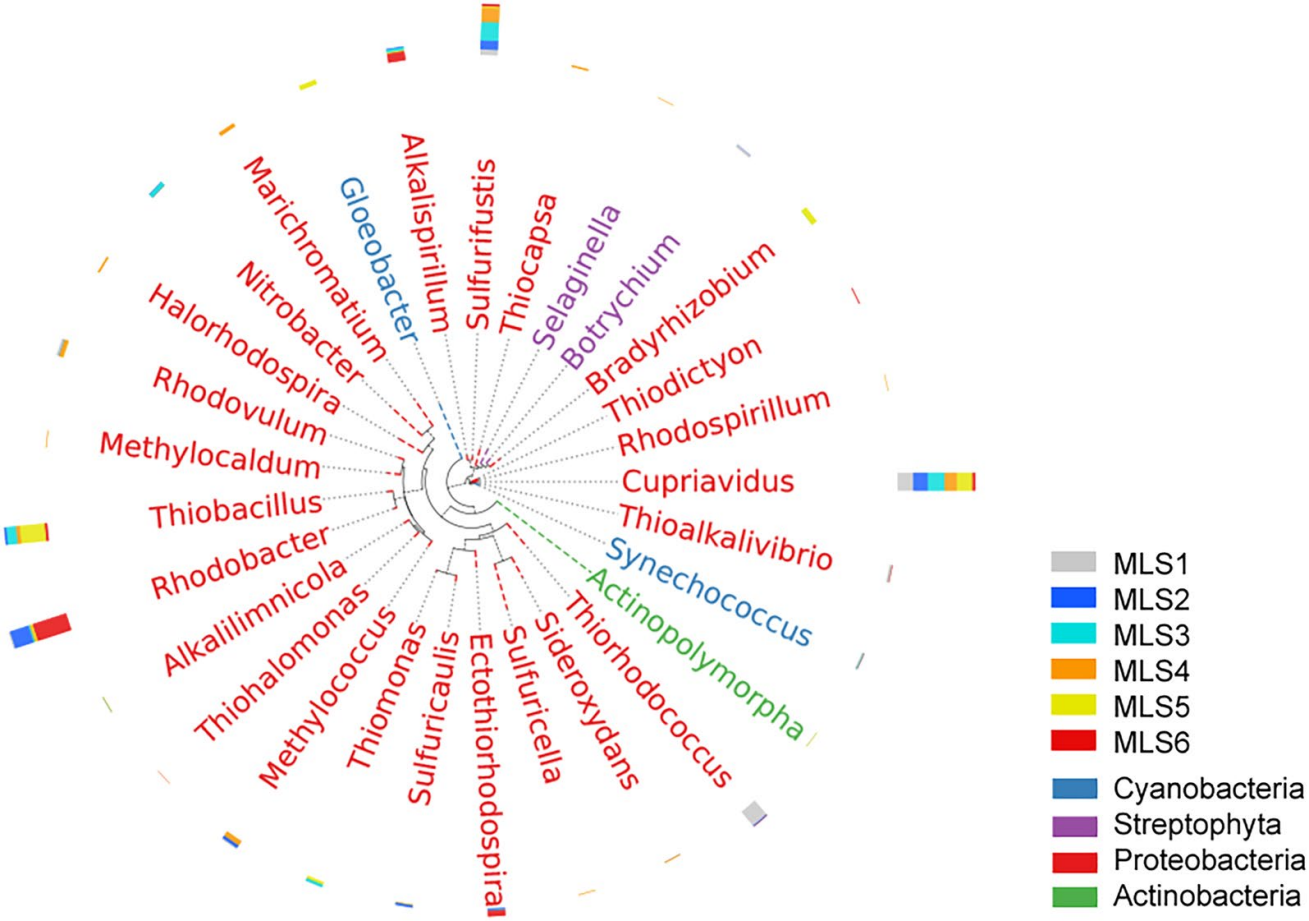
Horizontal evolutionary tree of the top 30 genera of OTU abundance of CO_2_-fixing microbes along altitude gradient on the Mila Mountain.

The representative sequence corresponding to the top 30 abundant OTUs was selected to build the evolutionary tree in the unit of the genus (Fig. 3). In the figure, it could be found that the abundant OTU can be divided into four phyla. Proteobacteria was predominant. Cyanobacteria, Actinobacteria, and Streptophyta, constitute a small proportion. The most abundant bacteria are *Cupriavidus*, *Rhodobacter*, *Sulfurifustis* and *Thiobacillus*.

### Correlation between soil carbon fixing microbial community composition and environmental factors

**Figure 4:**
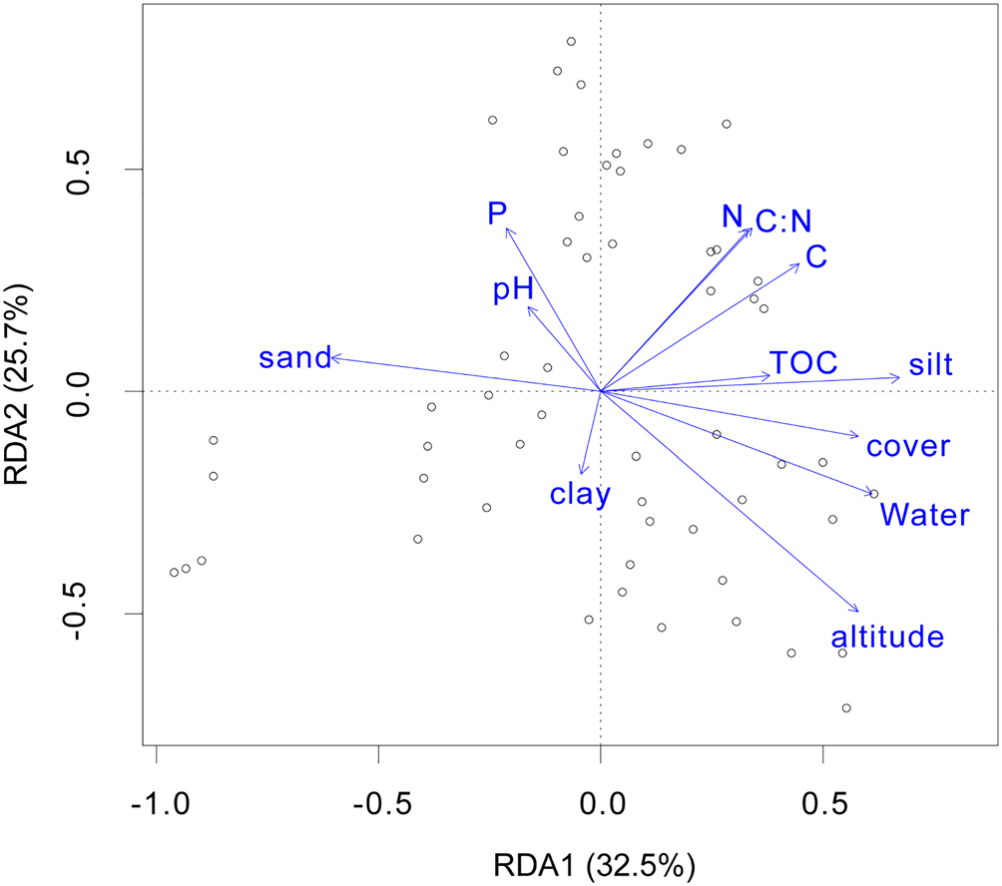
Redundancy analysis between soil microbial diversity and environmental factors.

Through redundancy analysis (RDA) can draw the correlation between soil CO_2_- fixing microbial community composition and environmental factors in Mila Mountain (Fig. 4). The arrow represents different environmental factors, the angle represents the correlation between the two environmental factors, and the ray length of environmental factors represents the influence degree of the influencing factors. It shows that RDA1 and RDA2 components accounted for 58.2% of the variation in community structure. Altitude has the greatest influence on the structural characteristics of CO2-fixing microbes, and other environmental factors are significantly correlated with altitude. Therefore, altitude influences the structural characteristics of CO2-fixing microbes by driving environmental factors.

## Discussion

This study found that Proteobacteria dominate in carbon-fixing microorganisms. Proteobacteria has also been found to be dominant in different habitats^24–28^; this may be due to the low habitat specificity of Proteobacteria, and strong ability to adapt to different environments^29^. Among them, the most abundant bacterial genera are *Cupriavidus*, *Rhodobacter*, *Sulfurifustis* and *Thiobacillus*. We also found a small number of cyanobacteria, Actinobacteria. As primary producers and predators in the ecological food chain, cyanobacteria are widespread in the natural environments^30–32^. Actinobacteria are mainly involved in the decomposition of organic matter^33^, which plays an important role in accelerating the decomposition of animal and plant residues in the soil, and also promotes the soil nitrogen cycle^34^.

We found that the diversity of carbon-fixing microorganisms in Mila Mountain showed a mid-peak similar to flora and fauna as the altitude increased. At MLS4 on the east slope (4500–4700 m), the Chao 1 index and Shannon diversity index of soil carbon-fixing microorganisms are much higher than those of other altitude gradient groups, and the overall OTU abundance first increases and then decreases with the elevation and mainly concentrated in the altitude gradient of 4300–4900 m. However, in many previous studies soil microbial diversity showed a monotonous decreasing trend with increasing altitude^35–38^. The reason why our results are inconsistent may be related to the content of soil organic carbon. Studies have shown that soil organic carbon has a significant impact on the carbon-fixing microbial community structure^39^. In addition, the Chao 1 index and Shannon diversity index of the east slope as a whole is generally higher than that of the west slope. Due to the differences in temperature and precipitation in different slopes, the microbial diversity also exhibited different altitude distribution patterns^22^.

As the altitude gradient rises, environmental factors such as soil and climate change, causing changes in the diversity of soil microbial communities. Therefore, changes in altitude gradients are an ideal natural laboratory for studying the characteristics of soil carbon-fixing microbial communities and their driving factors^16^. Soil parameters have a strong correlation with altitude, and the most significant correlation with altitude gradient is soil moisture content^41^. Relevant studies have shown that soil water content is very important in regulating microbial activity and diversity. It directly affects the physiological state of microorganisms, restricts the ability of microorganisms to decompose certain compounds, and regulates soil enzymes and soil physical and chemical properties, thereby affecting microbial composition and activity^41^. Guo et al. (2015) found that RubisCO enzyme catalyzing the Calvin cycle was promoted by SWC and NH_4_^+^ and inhibited by temperature^16^. Autotrophic organisms are usually controlled by temperature, plateau region is rich in microorganisms, this may be the region of microbial growth in this environment for a long time, can endure or adapt to low temperature. At the same time, the soil water content may counteract the inhibition of low temperature on microbial and enzyme activities. Mila Mountain connects the high mountain valleys of southeast Tibet and southwest Tibet. The climate on the east and west slopes are quite different. The eastern part is rich in precipitation and belongs to a warm and semi-humid climate type; the western part is dominated by a warm and semi-arid climate. Previous research indicated that the slope aspect markedly affects soil and microbiological properties in micro-ecosystem environments. We also found that there are differences in microbial communities on the eastern and western slopes. It may be that the slope aspect changes the microclimate of the study area, which determines the amount of solar radiation absorbed by the soil, thus affecting the soil water content and soil temperature and then affecting the soil microbial content and community structure. Similarly, Sidari et al. (2008) also found that the difference of soil microbial biomass in different slope aspects may be caused by the different microclimate under this condition^41^.

## Materials and methods

### Site description and sample collection

The study area is located in the Mila Mountain of the Tibetan Plateau, connecting the high mountain valleys of southeast and southwest Tibet. The eastern part is rich in precipitation and belongs to a warm and semi-humid climate type; the western part is dominated by a warm and semi-arid climate. Vertically, Mila Mountain includes mountain temperate, subalpine cold temperate, and alpine cold temperate climate zones. The structure and distribution pattern of vegetation communities are affected by different climate types and soil types on the east and west slopes.

**Figure 5:**
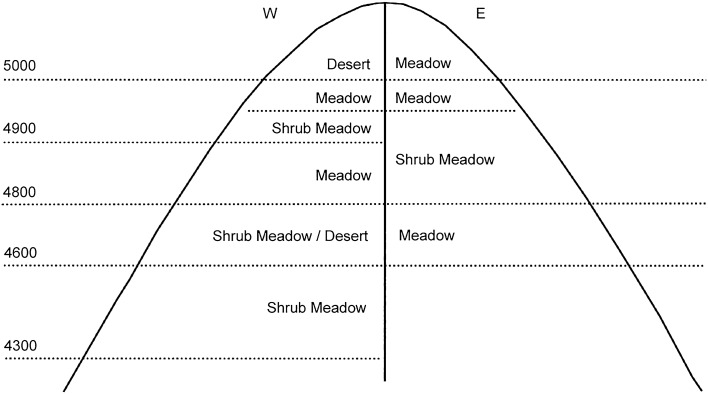
Schematic diagram of vegetation distribution with different altitude gradients on the eastern and western slopes of Mila Mountain^42^.

In late July 2017, soils were sampled from both slopes and mountaintop of Mila Mountain region with five replicates at six altitude gradients (3900–4100 m; 4100–4300 m; 4300–4500 m; 4500–4700 m; 4700–4900 m; 5000–top). Vegetation distribution on the eastern and western slopes of Mila Mountain with different elevations as shown in the Fig. 5. Five sampling plots are arranged symmetrically on the east, and west slopes along the altitude gradient, and a single sampling plot are set on the top of the mountain. The five-point sampling method is adopted platform sampling. At each plot, five surface soil samples (0–20 cm) were collected, and a total of 55 samples were collected. After the samples were collected, they were stored in a refrigerator with blue ice at − 80 °C and then transported back to the laboratory for DNA extraction and subsequent experiments. The remaining soils were air-dried for physicochemical analyses.

### Analysis of soil physicochemical properties

The soil moisture content was determined by the drying method. Soil pH was determined with PHS-3C by the potentiometric method. The soil particle size was measured with Mastersizer 3000 laser particle size analyzer. Soil total carbon and organic carbon are measured by multi-C/N310 host and HT1300 solid module.

### DNA extraction

Soil microbial DNA was extracted by E.Z.N.A.™ Soil DNA Kit. The soil sample was homogenized and then processed in a specially formulated buffer containing detergent. Humic acids, proteins, polysaccharides and other contaminants were subsequently precipitated after the thermal freezing step. Extraction was performed, the binding conditions were adjusted, and the sample was applied to the HiBind™ DNA spin column. Trace contaminants were removed by two rapid washing steps, and pure DNA was eluted in water or a low ionic strength buffer. Purified DNA can be used directly for subsequent analysis.

### PCR amplification and MiSeq library

After the genomic DNA extraction is completed, the sample is melted on ice, centrifuged and mixed thoroughly; Nanodrop detects the quality of the sample and takes 30 ng for PCR amplification. According to the designated *cbbL* 2 amplification region, specific primers with barcode or synthesize fusion primers with misplaced bases were synthesized. KAPA 2G Robust Hot Start Ready Mix and *cbbL* 2 primers were adopted for PCR amplification. 1% agarose gel electrophoresis was used to detect the extracted genomic DNA.

According to the pooling ratio, it was pooling a certain volume of the PCR product of the fusion primer without misplaced bases into a computer library and using 2% agarose gel to screen the library fragments. The selected library fragments were detected and quantified by Qubit. A certain amount of library was added to 10 μl Endrepair&Add A for end repair and A tailing Then 33.5 μl Adaptor Ligation Mix was added for connection with sequencing adapters, and the library was purified and recovered. Then add linker primers, enzymes, and Mix for PCR enrichment to complete library construction. Finally, the library was purified by the magnetic bead method. Use Nanodrop to roughly check library concentration, Agilent 2100 to detect library fragments. The library was sequenced on the Illumina Miseq platform.

### Statistical analysis

In MEGA 6.0 software, representative sequences of the top 30 OTUs corresponding to abundance were selected by genus to construct phylogenetic trees. The formula of the Vegan package in R (V.3.5.2) was used to conduct analyze community structure and community diversity. They were drawing using Adobe Illustrator 2020 software.

## Conclusion

Our results revealed the community structure of carbon-fixing microorganisms and their influencing factors along an altitude gradient. In the Mila Mountain area, the overall soil OTU abundance was concentrated at 4300–4900 m, showing a mid-peak trend. Proteobacteria dominated the soil carbon fixing microbial community and might play an important role in the CO_2_ fixation in alpine meadow soils. The relatively abundant bacteria belong to *Cupriavidus*, *Rhodobacter*, *Sulfurifustis*, and *Thiobacillus*. Within the elevation gradient in this study, the abundance and community structure of carbon-fixing microorganisms in meadow soil was mainly affected by the elevation change.

## Supplementary Information

Supplementary Table S1.
