# Supplementary material for: Diversity and distribution of CO2-fixing microbial community along elevation gradients in meadow soils on the Tibetan Plateau
Source: Sci Rep. 2022 Jun 10;12:9621. doi: 10.1038/s41598-022-13183-4 (PMC9187700; doi:10.1038/s41598-022-13183-4)
Supplement: Supplementary file 1 — Supplementary Table S1. [file 41598_2022_13183_MOESM1_ESM.docx]

Table S1 Soil physic-chemical properties. (Mean±SD)

|  | Sample  site | Altitude  m | Organic carbon content  g/kg | Total nitrogen  g/kg | Total carbon  g/kg | Available phosphorus  mg | Moisture content  % | pH | Sand content  % | Clay content  % |  |
| --- | --- | --- | --- | --- | --- | --- | --- | --- | --- | --- | --- |
|  |  |  |  |  |  |  |  |  |  |  |  |
|  |  |  |  |  |  |  |  |  |  |  |  |
| Eastern slope | MLSD1 | 3875 | 49.55±2.87 | 0.54±0.17 | 6.4±1.68 | 5.61±0.98 | 24.46±3.48 | 5.59±0.05 | 28.57±4.67 | 5.82±1.38 |  |
|  | MLSD2 | 4121 | 56.16±19.98 | 0.57±0.12 | 6.73±1.33 | 6.07±1.93 | 31.03±9.91 | 5.34±0.17 | 28.62±7.33 | 5.57±1.93 |  |
|  | MLSD3 | 4313 | 63.62±12.29 | 0.68±0.27 | 7.46±2.33 | 9.2±1.65 | 32.9±6.12 | 5.36±0.36 | 25.73±3 | 3.8±0.45 |  |
|  | MLSD4 | 4515 | 55.11±7.39 | 0.38±0.05 | 4.94±0.6 | 6.73±1.33 | 42.59±4.23 | 5.33±0.39 | 33.45±2.97 | 3.27±0.38 |  |
|  | MLSD5 | 4725 | 48.14±6.91 | 0.29±0.04 | 4.02±0.51 | 3.37±1.27 | 37.63±2.95 | 5.34±0.69 | 21.77±6.29 | 5.59±0.88 |  |
| Mountaintop | MLS6 | 5020 | 41.42±7.11 | 0.37±0.04 | 5.04±0.76 | 2.56±0.35 | 30.22±9.27 | 5.6±0.22 | 21.93±1.54 | 7.62±0.63 |  |
| Western slope | MLSX5 | 4846 | 50.5±6.57 | 0.41±0.12 | 4.94±1.53 | 4.15±1.76 | 38.82±5.39 | 5.07±0.32 | 21.81±1.78 | 6.88±0.52 |  |
|  | MLSX4 | 4513 | 55.08±18.78 | 0.36±0.08 | 4.61±0.86 | 4.57±0.71 | 32.26±1.24 | 4.99±0.38 | 23.52±1.51 | 6.58±0.85 |  |
|  | MLSX3 | 4377 | 49.67±15.19 | 0.23±0.03 | 2.89±0.52 | 5.05±0.46 | 21.31±2.81 | 6.05±0.74 | 36.52±4.76 | 5.68±0.99 |  |
|  | MLSX2 | 4145 | 29.31±3.83 | 0.25±0.05 | 2.89±0.77 | 2.56±0.46 | 23.85±5.7 | 5.72±0.2 | 32.24±0.64 | 5.66±1.01 |  |
|  | MLSX1 | 3867 | 26.17±3.12 | 0.24±0.03 | 2.88±0.76 | 2.87±0.81 | 12.75±2.55 | 5.48±0.35 | 37.5±4.46 | 6.98±1.52 |  |
